# Black Cohosh: An Uncommon Culprit of Bradycardia in Postmenopausal Women

**DOI:** 10.7759/cureus.55984

**Published:** 2024-03-11

**Authors:** Ahmad W Haddad, Adeniyi Adelakun, Wassim Abouzeid, Noreen Mirza, Dilesha Kumanayaka, Deema W Haddad, Joaquim Correia

**Affiliations:** 1 Internal Medicine, Saint Michael's Medical Center, Newark, USA; 2 Internal Medicine, St. Mary's General Hospital, Passaic, USA; 3 Cardiology, Saint Michael's Medical Center, Newark, USA; 4 College of Medicine, Jordan University of Science and Technology, Irbid, JOR

**Keywords:** left axis deviation., sinus bradycardia, post-menopause, heart arrythmia, cardiac arrythmia, drug induced bradycardia

## Abstract

The use of herbal supplements has become increasingly prevalent, with black cohosh (BC) gaining popularity for managing menopausal symptoms. However, reports of adverse effects associated with BC are limited. We present a case of bradycardia linked to prolonged BC ingestion. A 76-year-old postmenopausal woman who has been taking BC for years has had syncopal attacks multiple times during the past years associated with bradycardia with the heart rate dropping to 30 beats/minute with no identifiable cause. Discontinuation of BC resulted in a gradual resolution of bradycardia, highlighting a plausible association. There were no additional pharmacological or invasive interventions required.

## Introduction

*Actaea racemosa*, also known as *Cimicifuga racemosa*, is a plant native to North America and belongs to the buttercup family. It is a natural source of a popular herbal remedy known as black cohosh (BC), often used to alleviate menopausal symptoms due to its estrogen-like properties [1]. Breast soreness, uterine bleeding, and musculoskeletal symptoms were among the adverse events noted. The most frequent negative effects reported by users who have taken big amounts are rashes and gastrointestinal discomfort. The less frequent adverse effects are nausea, vomiting, and dizziness. However, items marketed as "black cohosh" have been connected to multiple incidents of acute liver damage in recent years. Some of these cases have been so bad that they have killed the patient or necessitated an urgent liver transplant [1,2]. Despite its widespread use, there is still a lack of documented adverse effects associated with BC. Here, we present a case of bradycardia in a postmenopausal woman, which is an uncommon adverse reaction linked to the ingestion of BC. This is the first reported case in the literature of asymptomatic bradycardia in a patient who is taking BC.

## Case presentation

A 76-year-old Hispanic female with a history of hypertension, hyperlipidemia, and provoked pulmonary embolism after a fall seven years ago presented to the ED after a syncopal episode, during which time she was unconscious for a few seconds in a sitting position. She was not on anticoagulation at the time. On arousal, she endorsed sudden onset dizziness, increased warmth, and palpitations prior to the syncopal episode. The patient also stated that she had a similar event while at a hair salon a year ago and did not seek any medical evaluation at the time. The patient’s home medications were reviewed, which included low-dose aspirin, moderate-intensity atorvastatin, spironolactone, and amlodipine. She also reported she was started on BC 6.5-160 milligrams twice a day almost 10 years ago by her gynecologist for long-standing post-menopausal hot flashes, and she is regularly following up with her every two to three years. She recommended her to stop taking BC, but she feels better with it.

Her initial vital signs included a temperature of 97.7 degrees Fahrenheit, heart rate of 51 beats/minute, blood pressure of 163/82 mmHg, respiratory rate of 20 breaths per minute, and SPO_2_ of 100% on room air. Orthostatic blood pressure was negative. On examination, she was alert and oriented to time, place, and date, and the cardiac exam revealed normal S1/S2 and bradycardia with a heart rate in the 50s. There were no murmurs, rubs, or gallops. Her lung exam revealed clear bilateral air entry with no skin manifestations, and she denied any feeling of palpitations when she was evaluated in the ED.

Initial laboratory showed a normal chemistry panel and complete blood count with hemoglobin of 13.5 g/dL with a normal white cell differential. High-sensitivity troponin trended from 33 to 96, to 107, and later to 98. Thyroid-stimulating hormone (TSH) was normal at 1.097, with total creatinine kinase (CK) of 75 U/L and no chest pain or palpitations at this time.

**Figure 1:**
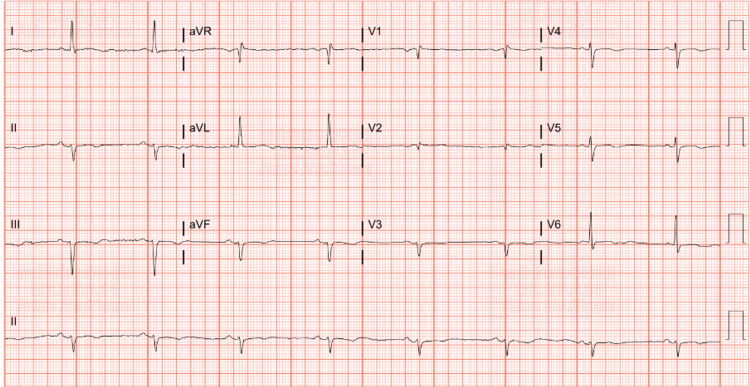
ECG on admission revealed sinus bradycardia with a heart rate of 47, left axis deviation, and poor R-wave progression.

An electrocardiogram revealed sinus bradycardia with a heart rate of 47, left axis deviation, and poor R-wave progression with QTc of 517 (Figure 1). A transthoracic echocardiogram revealed a normal ejection fraction of 60% with no valvular abnormalities. Initial telemetry monitoring of the heart rate and rhythm recorded baseline sinus bradycardia in the 50s, with episodes, especially at night, running in the 30s, lasting for up to one minute, and with no episodes of heart block or abnormal rhythm. As the patient's care progressed over four days, computed tomography angiographies (CTAs) of the coronaries and chest were done, with a nuclear test, and all came back negative with no evidence of pulmonary embolism or ischemic changes in the myocardium. As she was being observed and evaluated for the need for a pacemaker by the electrophysiology team, her heart rate improved to baseline in the 60s. BC was not continued during admission, and she did not complain of any episodes of hot flashes. She was advised to follow up with her obstetrician-gynecologist after two weeks of the discharge. She was also instructed to follow up with the cardiology clinic to monitor any episode of palpitations or dizziness. At the time of the follow-up, she stopped taking her BC, and she denied any episode of palpitations.

## Discussion

Sinus bradycardia when having <60 beats per minute. Nonetheless, 15% of Asians in good health have a heart rate that is less than 60 beats per minute, with 18% of men and 9% of women having this condition [3]. Usually, bradycardia is asymptomatic. On the other hand, syncope and the development of new heart failure or worsening of preexisting heart failure are severe consequences of certain types of sinus bradycardia [4]. Furthermore, compared to 60-100 beats/min, the mortality rate increases at a heart rate of less than 50 beats/min [5]. Therefore, in clinical practice, sinus bradycardia needs to be taken seriously. According to the most recent guidelines from the American College of Cardiology/American Heart Association, one of the main indications for pacemaker implantation is clinically significant symptomatic bradycardia [6]. Pacemaker implantation is often deemed unnecessary; nevertheless, if symptomatic bradycardia is drug-induced, the condition is predicted to recover upon withdrawal of the offending medicine [6]. There are many medications and other components that can be a culprit leading to bradycardia. Most of them were described in a scientific statement from the American Heart Association, such as antiarrhythmics (flecainide), anticancer (thalidomide), antidepressant (citalopram), and antihypertensive (clonidine, β-blockers (including eye drops), diltiazem, and verapamil) medications [7].

*Actaea racemosa*, or isopropanol *Cimicifuga racemosa* root extract, is commonly called BC. It has been extensively investigated, and the BC formulation is the most well-researched [8]. Historically, Native Americans in the eastern United States and Canada used BC to treat ailments, such as rheumatism, menstrual irregularities, sore throats, malaria, and discomfort during childbirth [9]. There has been growing interest in using it to alleviate menopausal symptoms. A recent systematic review revealed that the rate of adverse events was approximately 5.4%. Out of these, over 97% of the identified events were minor. The review discovered that most adverse events were related to gastrointestinal symptoms and musculoskeletal and connective tissue disorders [10]. Recent reviews have analyzed the most frequently reported significant adverse event linked to BC hepatotoxicity [10]. Other severe side effects that have been documented include myotoxicity, cutaneous vasculitis, and anaphylaxis [2,11,12].

The exact method by which BC works is yet to be discovered. Several biologically active components may be found in the BC rhizome, such as long-chain fatty acids, resins, caffeic acids, ferulic acids, phytosterols, fumarolic acid, salicylic acid, sugars, and tannins [13]. Triterpene glycosides activity, 27-deoxyactein, and cimicifugoside are also present. With BC preparations, serotonin selective reuptake inhibition is not the cause of the serotonergic effects that have been seen. BC is a partial agonist at serotonin receptors and demonstrates competitive binding to the 5-HT1A, 5-HT1D, and 5-HT7 receptors [14,15]. This is significant since research indicates that the hypothalamus' ability to suppress a rise in blood pressure and heart rate is inhibited when 5-HT1A receptors are activated [16]. An explanation for how BC mediates bradycardia has not been easily found in the published pharmacology of its constituent parts. Notably, bradycardia is one of the side effects of BC most frequently listed in non-academic literature, and investigations on animals have shown that administering its extracts can cause significant bradycardia [2]. One case in the literature mentioned heart block as a clinical side effect of this component [17]. In our patient, she had sinus bradycardia, which ultimately resolved after she stopped taking BC with no need for pacemaker implantation. The bradycardia was reversible, and the cause was discovered.

## Conclusions

This case highlights the significance of being aware of herbal supplements as a potential source of adverse cardiac outcomes. The mechanism of action of BC on the cardiovascular system is still unclear, and further research is needed to understand it better. Healthcare professionals should be cautious and have an open discussion about the use of herbal supplements, particularly with patients who have pre-existing cardiovascular conditions.

## References

[1] Muqeet Adnan M, Khan M, Hashmi S, Hamza M, AbdulMujeeb S, Amer S (2014). Black cohosh and liver toxicity: is there a relationship?. Case Rep Gastrointest Med.

[2] Borrelli F, Ernst E (2008). Black cohosh (Cimicifuga racemosa): a systematic review of adverse events. Am J Obstet Gynecol.

[3] Wu J, Kors JA, Rijnbeek PR, van Herpen G, Lu Z, Xu C (2003). Normal limits of the electrocardiogram in Chinese subjects. Int J Cardiol.

[4] Ovsyshcher IE, Barold SS (2004). Drug induced bradycardia: to pace or not to pace?. Pacing Clin Electrophysiol.

[5] Liu EF, Chen L, Gao BX (2012). Sinus bradycardia: normal phenomenon or risk factor? Evaluation based on recent evidence. J Insur Med.

[6] Gregoratos G, Abrams J, Epstein AE (2002). ACC/AHA/NASPE 2002 guideline update for implantation of cardiac pacemakers and antiarrhythmia devices: summary article: a report of the American College of Cardiology/American Heart Association Task Force on Practice Guidelines (ACC/AHA/NASPE Committee to Update the 1998 Pacemaker Guidelines). Circulation.

[7] Tisdale JE, Chung MK, Campbell KB, Hammadah M, Joglar JA, Leclerc J, Rajagopalan B (2020). Drug-induced arrhythmias: a scientific statement from the American Heart Association. Circulation.

[8] Briese V, Stammwitz U, Friede M, Henneicke-von Zepelin HH (2007). Black cohosh with or without St. John's wort for symptom-specific climacteric treatment--results of a large-scale, controlled, observational study. Maturitas.

[9] Leach MJ, Moore V (2008). Black cohosh (Cimicifuga spp.) for menopausal symptoms (protocol). Cochrane Database Syst Rev 2008.

[10] Frempong W, Kiuru A, Ericsson J, Farah M. Cimicifuga racemosa L (69982009). Nutt. (black cohosh) and anaphylactic reactions, including face and oral oedema. https://www.who-umc.org/graphics/6998.pdf.

[11] Ingraffea A, Donohue K, Wilkel C, Falanga V (2007). Cutaneous vasculitis in two patients taking an herbal supplement containing black cohosh. J Am Acad Dermatol.

[12] Minciullo PL, Saija A, Patafi M, Marotta G, Ferlazzo B, Gangemi S (2006). Muscle damage induced by black cohosh (Cimicifuga racemosa). Phytomedicine.

[13] (2006). Therapeutic Goods Administration Adverse Drug Reactions Advisory Committee. Hepatotoxicity with black cohosh. Aust Adverse Drug React Bull.

[14] Powell SL, Gödecke T, Nikolic D (2008). In vitro serotonergic activity of black cohosh and identification of N(omega)-methylserotonin as a potential active constituent. J Agric Food Chem.

[15] Burdette JE, Liu J, Chen SN (2003). Black cohosh acts as a mixed competitive ligand and partial agonist of the serotonin receptor. J Agric Food Chem.

[16] Villela DC, da Silva LG Jr, Fontes MA (2009). Activation of 5-HT receptors in the periaqueductal gray attenuates the tachycardia evoked from dorsomedial hypothalamus. Auton Neurosci.

[17] McKenzie SC, Rahman A (2010). Bradycardia in a patient taking black cohosh. Med J Aust.

